# Metastasis Disproved by the Structure and Functions of Tissues

**Published:** 1847-07

**Authors:** 


ART. XI.—*Metastasi Riprovate dalla Struttura dei Tessuti e dalle funzioni dei Medesimi.* Tratto di G. B. BELLINI, Maestro Operatore in Santa Maria Nuova.—*Firenze,* 1845.

*Metastasis Disproved by the Structure and Functions of Tissues.* By G. B. BELLINI, Operating Surgeon in the large Hospital of Florence.—*Florence,* 1845.

OF this work one fasciculus only has reached us. Its object is to prove that what is generally considered to be metastasis is not so, but a new disease generated in the part supposed to be the seat of metastasis; that the functions of the absorbent and circulatory systems would explain the fact of morbid matter being taken from one part and affecting the general system, but not setting up a disease confined to one organ or tissue; that many supposed instances of metastasis are contemporaneous attacks in different parts, or mere extension of disease; that others are simplc instances of reflex nervous irritation, or excitement of some organ which has to do the duty of another, whose functions are interfered with by disease.

It would be unfair to criticise the reasoning of Dr. Bellini before the completion of his work. Our readers can form their own opinions of its nature and value from the statement we have just given of its objects.

